# Azugraphene: a new graphene-like hexagonal carbon allotrope with Dirac cones†

**DOI:** 10.1039/c9ra07953j

**Published:** 2019-10-28

**Authors:** Jing Liu, Haigang Lu

**Affiliations:** Key Laboratory of Materials for Energy Conversion and Storage of Shanxi Province, Institute of Molecular Science, Shanxi University Taiyuan 030006 P. R. China luhg@sxu.edu.cn

## Abstract

Classic two-dimensional graphene possesses outstanding properties due to Dirac cone structures so that many Dirac cone materials had been predicted. Using the first principle symmetric search algorithm, a new graphene-like carbon allotrope with *P*6̄2*m* space group, named azugraphene, was predicted and its 38 atoms in the unit cell can be fragmented into three 5–7 rings of azulene, one hexagon, and two remainder atoms. Azugraphene is a low-energy graphene-like hexagonal carbon allotrope with six Dirac cones in the first Brillouin zone. Due to its stability and the existence of its elementary fragments, azugraphene is potentially synthesizable. In addition, the stable AB stacking bilayer azugraphene is also a Dirac cone material with a band gap of 2.5 meV. Therefore, both the monolayer and bilayer azugraphenes have great potential in physics, chemistry, and nanoelectronics.

## Abstract 2

Azugraphene is a new graphene-like hexagonal carbon allotrope with Dirac cones and is potentially synthesizable from its fragments: azulene.

## Graphical abstract

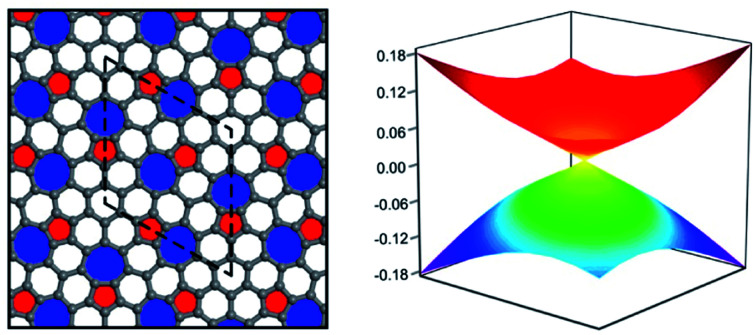

## Introduction

Graphene, a two-dimensional (2D) array of planar hexagonal units (C_6_) of sp^2^-hybridized carbon atoms, has attracted tremendous interest due to its extraordinary electrical, thermal, and physical properties.^1–4^ Enormous research attention has been devoted to the design and development of novel 2D carbon allotropes using both theoretical and experimental methods. Up to now, numerous 2D carbon allotropes were predicted with distinct electronic properties including metallic, semimetallic, semiconducting, and topologically insulating in nature. In addition, there are many predicted 2D carbon allotropes with exotic geometric structures, such as the penta-graphene composed of only carbon pentagons^5^ and the PCF-graphene with a finite thickness.^6^

Among all these 2D allotropes, the Dirac cone materials attract more attentions because of its extraordinary electrical properties. From 2012, a series of 2D carbon allotropes with Dirac cones were predicted using the first principle methods, such as α-graphdiyne,^7^ α-, β-, and 6,6,12-graphyne,^8^ 14,14,14-graphyne and 14,14,18-graphyne,^9^ S-, D-, and E-graphene,^10^ phagraphene,^11^ and α-2 graphyne.^12^ However, most of these allotropes are much unstable in thermodynamics so that their possible preparations and potential applications are greatly limited in experiment. Recently, a very stable 2D Dirac allotrope composed of 5–6–7 carbon rings, Stone–Wales graphene (SW-graphene),^13^ was predicted, which is only 141 meV per atom less stable than graphene.

Beside graphene, another 2D carbon allotrope, graphdiyne (graphyne with diacetylene groups) was successfully synthesized on the surface of copper *via* a cross coupling reaction using its elementary fragment, hexaethynylbenzene,^14^ though it is very unstable in thermodynamics. On the other hand, to find the more stable 2D carbon allotropes, one of the feasible bottom-up approaches is to mix some 5–7 rings into the hexagonal array. From 2015, a series of 5–6–7 2D carbon allotropes were predicted, such as phagraphene,^11^ twin graphene,^15^ ψ-graphene,^16^ C-57 carbon,^17^ which are generally more stable than the graphyne allotropes. However, most of them are no Dirac cones in their band structure, except that the *Cmmm* SW-graphene is a semimetal with distorted Dirac cones. Therefore, it is desirable to explore the stable 2D carbon allotropes with Dirac cones, just like the hexagonal graphene.

In this work, using the first principle symmetric search algorithm, we predicted a new 2D hexagonal carbon allotrope, which consists of 38 carbon atoms in its unit cell. This allotrope contains mainly three 5–7 rings of azulene to be named as azugraphene. As far as we know, azugraphene is a new stable graphene-like hexagonal allotrope with Dirac cones so that it has great potential in physics, chemistry, and nanoelectronics.

## Computational methods

The first principle symmetric search program were performed with 3–40 carbon atoms per unit cell.^18,19^ At first, five 2D unit cells were defined by *γ* = 120° and *a* = *b* = 4, 6, 8, 10, and 12 Å, respectively. Secondly, given a 2D groups *P*6, *P*6*mm*, *P*3, *P*3*m*1, or *P*31*m*, these predefined cells were filled by randomly putting the finite-sized atoms and their symmetric equivalent atoms step-by-step. In each step, all distances between two carbon atoms must be greater than 1 Å in its 3 × 3 supercell. At last, these filled cells were optimized using the first principle method.

The density functional theory calculations were performed by the VASP5.4.4 package^20,21^ using the projector augmented wave (PAW) method^22^ in conjunction with the PBE functional.^23^ The plane wave cut-off energy was 500 eV throughout our calculation. Each calculation was considered to be converged when the energy precision during the geometry optimization process were smaller than 10^−6^ eV per atom and the forces components on each atom were less than 0.01 eV Å^−1^. For geometric optimization, both the lattice constants and positions of atoms were fully relaxed. The structures is represented by a unit cell with a 15 Å vacuum region in the normal direction to avoid the interactions between neighboring layers. The Brillouin zone is sampled using *k*-points with 0.02 Å^−1^ spacing in the Monkhorst–Pack scheme^24^ for all calculations. The van der Waals interactions between the bilayer sheets are corrected by the many-body dispersion energy method (MBD@rsSCS) of Tkatchenko *et al.*^25,26^

Phonon calculations were performed using the supercell approach. Real-space force constants of supercells were calculated in the density-functional perturbation theory (DFPT)^27^ implemented in the VASP5.4.4 package, and phonon frequencies were calculated from the force constants using the Phonopy 1.13.2 code.^28,29^

## Results and discussion

### Geometric structure

**Fig. 1:**
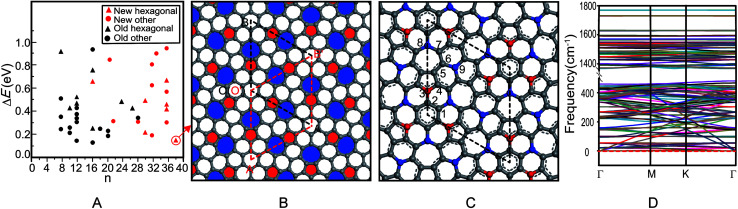
Relative stability of some 2D carbon allotropes (A), geometric structure (B), bonding pattern (C) and phonon spectra (D) of azugraphene. The “*n*” in A is the number of carbon atoms in its unit cell, the rhombus regions indicate the corresponding unit cells, and all spheres in B and C are carbon atoms.

The first principle symmetric search found 17 new 2D carbon allotropes, including some hexagonal structures and the other structures (Fig. 1A), in which the hexagonal azugraphene is the most stable one. As shown in Fig. 1B, the predicted azugraphene exhibits the *P*6̄2*m* space group with the lattice constant of 10.845 Å, which belongs to hexagonal symmetry similar to graphene. Because of the rotation–inversion axis 6̄, azugraphene has two alternative unit cells: A with the lattice vectors of 
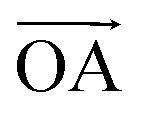
 and 
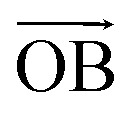
 (black rhombus), and A′ with the lattice vectors of 
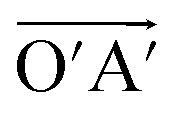
 and 
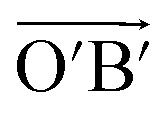
 (red rhombus). In each unit cell, their 38 atoms can be fragmented to three 5–7 rings of azulene (3 × 10 atoms, red and blue rings), one hexagon (6 atoms), and two remainder atoms. In the unit cells of A and A′, the orientations of all 5–7 rings are just reverse. In the following, we will mainly investigate the unit cell A in detail.

As shown in Fig. 1C, the unit cell A contains nine chemically non-equivalent carbon atoms along its vertical edge: C1(0.135, 0.135, 0.500), C2(0.000, 0.127, 0.500), C3(0.000, 0.251, 0.500), C4(0.123, 0.384, 0.500), C5(0.078, 0.487, 0.500), C6(0.183, 0.632, 0.500), C7(0.142, 0.740, 0.500), C8(0.000, 0.729, 0.500), and C9(0.333, 0.667, 0.500), respectively. Contrast with the graphene, the charges of carbon atoms in azugraphene are not equal zero because of the existence of 5–7 carbon rings. The normalized population analysis of atomic orbital projection shows that most of atoms are approximately neutral charged (from −0.10*e* to +0.10*e*, grey spheres), and some atoms have considerable charges: C3 atoms (−0.21*e*, red spheres), C8 and C9 atoms (+0.18*e* and +0.17*e*, blue spheres). Therefore, the C3 atoms should be the promising nucleophilic sites, and the C8 and C9 ones should be the promising electrophilic sites in chemical reactions. Contrast with the unreactive graphene, azugraphene has many ordered chemical active sites to be an interesting chemical template.

According to the typical C–C lengths of the single (1.54 Å), double (1.34 Å), and aromatic (1.42 Å) bonds, the bonding pattern of azugraphene is also plotted in Fig. 1B. It shows that there are three classes of C–C bonds in azugraphene: (1) the aromatic bonds just like that in graphene, such as C1–C2 (1.422 Å), C3–C4 (1.387 Å), C4–C5 (1.427 Å), C5–C6 (1.401 Å), C6–C7 (1.450 Å); (2) the double bonds, such as C2–C3 (1.351 Å), and (3) the single bonds, such as C7–C8 (1.486 Å), C6–C9 (1.482 Å). Therefore, the existence of C–C double bonds in azugraphene implies that the azugraphene is more active than graphene in chemical addition reactions.

Because there is no imaginary mode in the phonon spectra of azugraphene in the entire Brillouin zone (Fig. 1D), azugraphene is completely planar in the ground state. Its highest frequency phonon mode at 1771 cm^−1^, distinctly larger than about 1600 cm^−1^ for graphene.

Among all hitherto reported 2D carbon allotropes, graphene has the lowest energy to be set as zero (Table S1†). Azugraphene is only 157 meV per atom less stable than graphene, and is 16 meV per atom less stable than the SW-graphene to be the third most stable one. The other 2D allotropes are less stable than azugraphene. The *Cmmm* SW-graphene^13^ is not similar to the hexagonal graphene in symmetry. Therefore, it is safe to predict that azugraphene is a new stable graphene-like hexagonal carbon allotrope, as shown in Fig. S1.†

In experiment, graphdiyne had been synthesized from its elementary fragment: hexaethynylbenzene. The main fragment of azugraphene is the 5–7 ring of azulene, an isomer of naphthalene, so that azugraphene should be synthesized from azulene. Because of its high stability and its existing elementary fragment, azugraphene is potentially synthesizable.

### Electronic properties

**Fig. 2:**
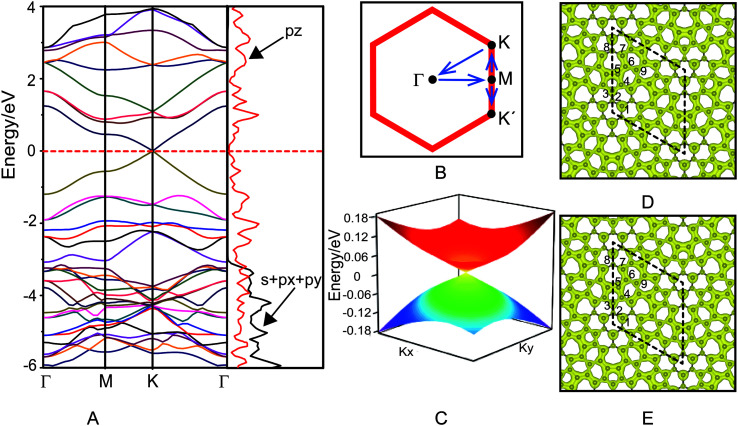
Band structure and projected density of states of azugraphene (A), *k*-path (B), three-dimensional band structure at the Dirac point (C), charge densities of valence band (D) and conduction band (E). The Fermi level is set as 0 eV in all cases.

The electronic structure of azugraphene is presented in Fig. 2. The band structure is plotted along the high symmetry *k*-points: Γ(0.00, 0.00, 0.00) → M(0.50, 0.00,0.00) → K(0.33, 0.33, 0.00) → Γ(0.00, 0.00, 0.00) (Fig. 2B). It can be seen from Fig. 2A that the energies of valence and conduction bands (VB and CB) are degenerate at a single point on the Fermi surface. This degenerate point is located at a high-symmetric *k*-point (K) within the first Brillouin zone. In particular, these bands are linearly dispersed with the *k*-vector in the proximity of the Fermi level. The valence and conduction bands meet the degenerate point with a slope (∂*E*/∂*k*) of ±25.2 eV Å along a line that passes through the high symmetric point K along both M and Γ directions. The 3D band structure of azugraphene was plotted on an energy scale of −0.18 to 0.18 eV (Fig. 2C). It suggests that the energy of Fermions (electrons and holes) is dispersed in the form a double cone near to the Fermi level within the first Brillouin zone. These features confirmed that the degenerate point of VB and CB on the Fermi level can be considered as the Dirac point. There are six Dirac points on the Fermi surface at the high-symmetric K and K′ points in the hexagonal Brillouin zone of azugraphene. Computed using the *E*(*q*)/*ħ*|*q*| relation, the Fermi velocity of azugraphene is 6.09 × 10^5^ m s^−1^, which is comparable to those of other 2D carbon allotropes, such as graphene (8.22 × 10^5^ m s^−1^),^2^ α-graphyne (6.77 × 10^5^ m s^−1^),^8^ β-graphyne (3.87–6.77 × 10^5^ m s^−1^),^8^ phagraphene (3.43–6.48 × 10^5^ m s^−1^),^11^ and α-2 graphyne (6.36–7.13 × 10^5^ m s^−1^).^12^

In order to understand the orbital contributions for the formation of linear band dispersion in the proximity of the Fermi level, the projected density of states (PDOS) were plotted in Fig. 2A. The PDOS on s + p_*x*_ + p_*y*_ of azugraphene disappears at about −3.0 eV, and the PDOS on p_*z*_ gradually decreases to zero around the Fermi level. This feature corroborates that the azugraphene is semimetallic in nature. It is evident from the band decomposed charge densities (Fig. 2D and E) of VB and CB that the p_*z*_ orbitals of carbon atoms are mostly responsible for the formation of linearly dispersed VB and CB in the proximity of the Fermi level, akin to that of graphene and graphynes. In particular, the p_*z*_ bonding and antibonding orbitals of carbon atoms in 5–7 rings are predominantly responsible for the formation of linear band dispersion nearer to the Fermi level, because these atoms are connected mainly by the aromatic bonds, as shown in Fig. 1C. And the C2 atoms in hexagons and C8 atoms in 7-rings have little contribution for the VB and CB, because these atoms are mainly taken part in the non-aromatic bonds.

### Bilayer azugraphene

**Fig. 3:**
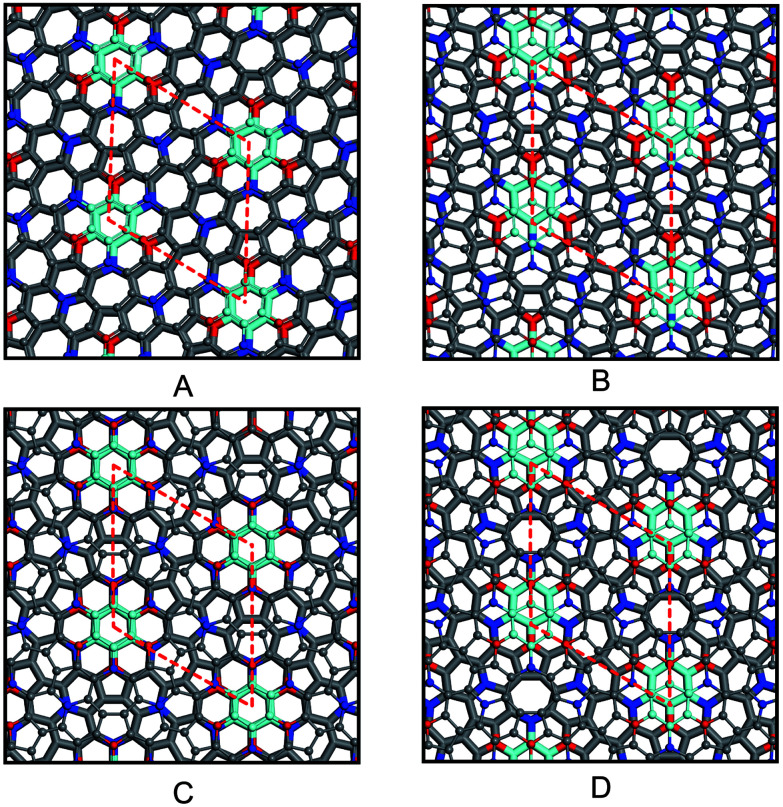
AA (A), AB (B), AA′ (C), and AB′ (D) stacking bilayer azugraphenes.

Because azugraphene has two alternative unit cells of A and A′, its bilayer sheets have four stacking modes: AA, AB, AA′, and AB′ (Fig. 3A–D), in which B and B′ layers are obtained by moving the A and A′ layers along 
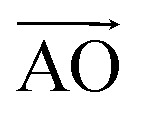
 directions one C–C bond length, respectively. Their interlayer binding energy were calculated by subtracting the energies of two monolayers from their corresponding energies of bilayers. The binding energies per atom of AA, AB, AA′, and AB′ are 20 meV, 24 meV, 21 meV, and 24 meV at the interlayer distances of 3.641 Å, 3.434 Å, 3.586 Å, and 3.447 Å, respectively. It is just as 20 meV per atom and 23 meV per atom of AA and AB stacking bilayer graphene. Consequently, there are two stable stacking bilayer azugraphene with the same stability, AB and AB′.

**Fig. 4:**
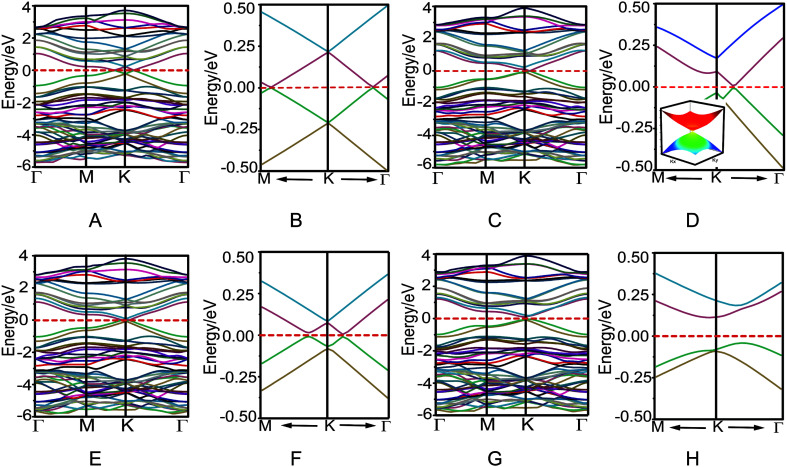
Band structures and their low-energy parts of AA (A and B), AB (C and D), AA′ (E and F), and AB′ (G and H) stacking bilayer azugraphenes. The inset of D is the 3D band structure of AB stacking bilayer azugraphene near the Dirac point.

The electronic properties of four bilayer azugraphenes were investigated and were compared with that of graphene. The low-energy bands of AA stacking bilayer azugraphene are linear and almost isotropic near the K-point (Fig. 4A and B). The interlayer coupling only makes its energy bands intersect each other and its Fermi energy changed from the K-point to the neighboring two points, akin to that of AA stacking bilayer graphene (Fig. S2C†). The low-energy bands of AB stacking bilayer azugraphene are linear near the K-points at the Γ direction (Fig. 4C and D) with a band gap of 2.5 meV, demonstrated by the 3D band structure of that plotted near the Dirac point. Therefore, the AB stacking bilayer azugraphene is also a Dirac cone materials, which is distinct with the AB stacking bilayer graphene (Fig. S2E†). The low-energy bands of AA′ stacking bilayer azugraphene are also linear near the K-point with a band gap of 12.2 meV (Fig. 4E and F). For the AB′ stacking bilayer azugraphene, the interlayer coupling obviously changes the azugraphene linear bands as the parabolic ones near the K-point (Fig. 4G and H), causing an indirect band gap (0.152 eV) between its valence and conduction bands to be semiconducting, which is different with the contact parabolic valence and conduction bands in the AB stacking bilayer graphene. Therefore, the bilayer azugrasphenes have two stable stacking forms: the Dirac cone material (AB) and semiconductor (AB′), which can be applied as the different electronic devices.

## Conclusions

Using the first principle symmetric search algorithm, a new graphene-like carbon allotrope with *P*6̄2*m* space group, azugraphene, was predicted. The 38 atoms in its primitive cell can be fragmented to three 5–7 rings (30 atoms), one hexagon (6 atoms), and two remainder atoms. Compared with the other 2D carbon allotropes, the azugraphene is a new stable graphene-like hexagonal one, and has six Dirac cones at the high symmetric K and K′ points in the Brillouin zone. In addition, the stable AB bilayer azugraphenes is also a Dirac cone materials with a band gap of 2.5 meV. Therefore, both the monolayer and bilayer azugraphenes have great potential in physics, chemistry, and nanoelectronics.

## Conflicts of interest

The authors declare that there is no conflict of interests.

## Supplementary Material

RA-009-C9RA07953J-s001
