# Supplementary material for: Azugraphene: a new graphene-like hexagonal carbon allotrope with Dirac cones
Source: RSC Adv. 2019 Oct 28;9(59):34481–5. doi: 10.1039/c9ra07953j (PMC9073862; doi:10.1039/c9ra07953j)
Supplement: RA-009-C9RA07953J-s001 [file RA-009-C9RA07953J-s001.pdf]

Electronic Supplementary Material (ESI) for RSC Advances.
This journal is © The Royal Society of Chemistry 2019

Azugraphene: a new graphene-like hexagonal carbon allotrope with Dirac cones

Jing Liu, Haigang Lu*

Institute of Molecular Science, Key Laboratory of Materials for Energy Conversion and Storage of Shanxi Province, Shanxi University, Taiyuan 030006, P. R. China.

*Correspondence should be addressed to Haigang Lu: luhg@sxu.edu.cn.

Supporting Information

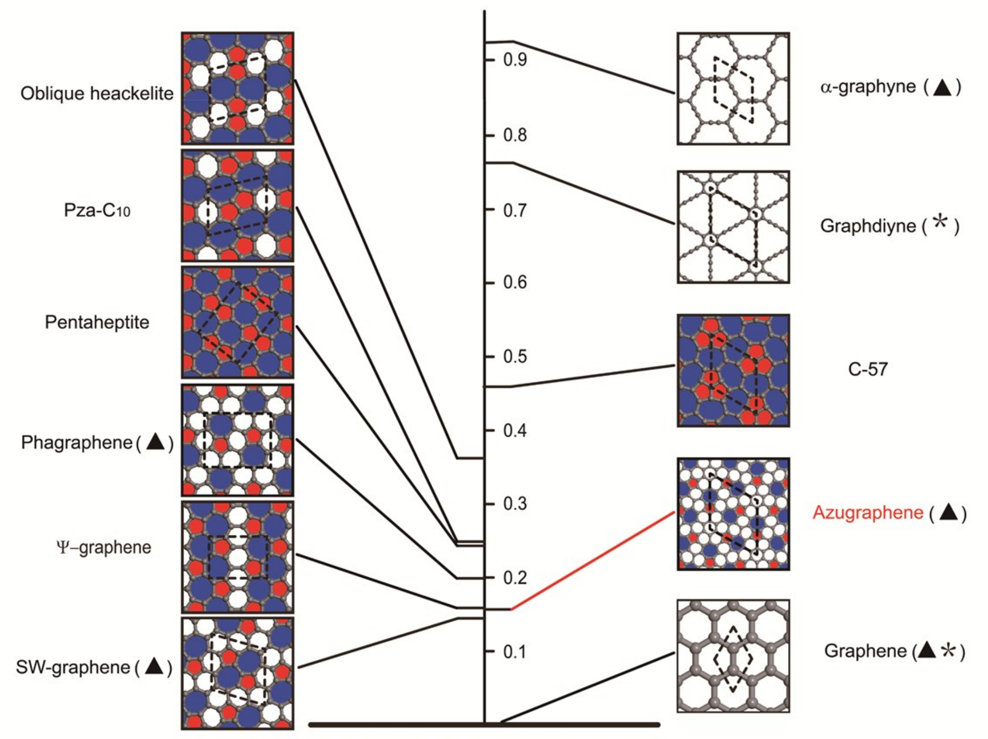

Figure S1. Energy profile of some important 2D carbon allotropes. The rhombus regions indicate the unit cells, the triangles indicates the Dirac cone materials, and the stars indicate the synthesized materials in the experiment.

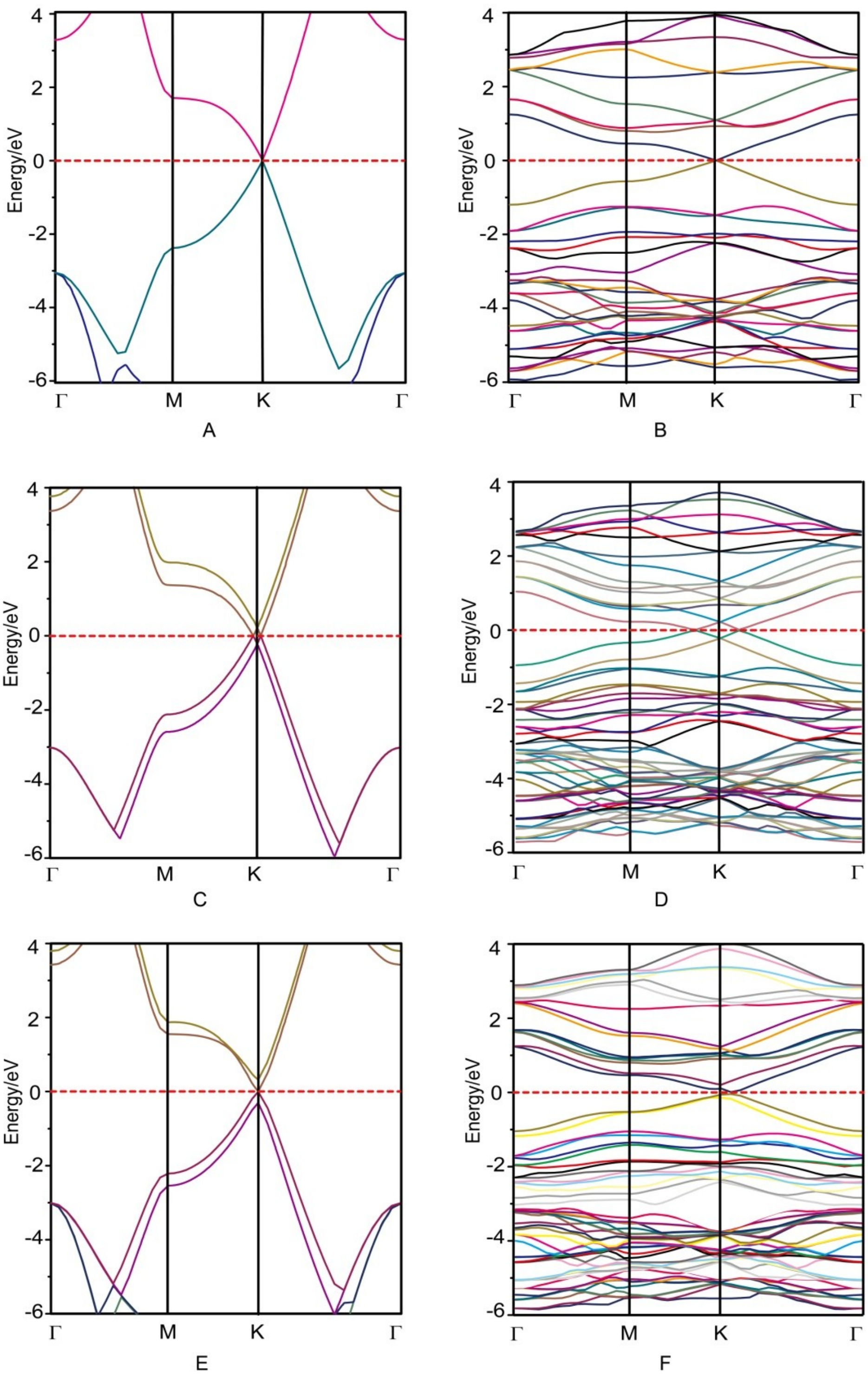

Figure S2. Band structures of graphene (A), azugraphene (B), AA stacking bilayer graphene (C), AA stacking bilayer azugraphene (D), AB stacking bilayer graphene (E), and AB stacking bilayer azugarphene (F). The Fermi energy (dashed lines) is set as zero.

Table S1. Space group, elementary polygons, lattice parameters *a* and *b* (Å), energy band gaps E_g (eV), and relative stable energy E_s (eV/atom) of some two-dimensional carbon allotropes.

Name	Space group	Elementary polygons	*a*	*b*	E_g	E_s
Graphene	$P6_3/mmc$	6	2.460		0	0
SW-graphene	Cmmm	5,6,7	6.683	6.683	0	0.141
Azugraphene	$P\bar{6}2m$	5,6,7	10.846		0	0.157
ψ-graphene	P2mg	5,6,7	6.696	4.844	-	0.158
Phagraphene	Pmg	5,6,7	8.110	6.642	0	0.199
Pentaheptite	Cmmm	5,7	7.472	5.867	-	0.243
Pza-C10	P2m	5,6,7	6.020	4.629	0.31	0.249
Oblique-Heackelite	P2m	5,6,7	5.529	6.091	-	0.362
C-57 carbon	$P\bar{6}2m$	5.7	6.144		-	0.459
graphdiyne	P6mm	6	9.421		0.46	0.763
α-graphyne	P6mm	18	6.966		0	0.921

Geometric structure of azugraphene (POSCAR of VASP):

```
1.00000
        9.39206     -5.42251       0.00000
        0.00000     10.84502       0.00000
        0.00000      0.00000      15.00000
     C
     38
Direct
   0.38369   0.12255   0.50000
   0.63153   0.18255   0.50000
   0.07797   0.48715   0.50000
   0.59820   0.85771   0.50000
   0.87744   0.26114   0.50000
   0.81744   0.44898   0.50000
   0.51284   0.59082   0.50000
   0.14228   0.74049   0.50000
   0.73885   0.61630   0.50000
   0.55101   0.36846   0.50000
   0.40917   0.92202   0.50000
   0.25950   0.40179   0.50000
   0.12255   0.38369   0.50000
   0.18255   0.63153   0.50000
   0.48715   0.07797   0.50000
   0.85771   0.59820   0.50000
   0.26114   0.87744   0.50000
   0.44898   0.81744   0.50000
   0.59082   0.51284   0.50000
   0.74049   0.14228   0.50000
   0.61630   0.73885   0.50000
   0.36846   0.55101   0.50000
   0.92202   0.40917   0.50000
   0.40179   0.25950   0.50000
   0.00000   0.86467   0.50000
   0.13532   0.13532   0.50000
   0.86467   0.00000   0.50000
   0.12651   0.00000   0.50000
   0.00000   0.12651   0.50000
   0.87348   0.87348   0.50000
   0.27065   0.27065   0.50000
   0.74895   0.74895   0.50000
   0.72934   0.00000   0.50000
   0.25104   0.00000   0.50000
   0.00000   0.72934   0.50000
   0.00000   0.25104   0.50000
   0.33333   0.66666   0.50000
   0.66666   0.33333   0.50000
```

Geometric structure of the other 16 new structures (POSCAR of VASP):

New structure 1.

```
    1.00000
      8.50874     -4.91252     -0.00000
      0.00000      9.82505     -0.00000
     -0.00000     -0.00000      9.94905
    C
    30
Direct
  0.91313   0.45656   0.50000
  0.14311   0.00000   0.50000
  0.70990   0.56610   0.50000
  0.70990   0.14379   0.50000
  0.28282   0.00000   0.50000
  0.54343   0.45656   0.50000
  0.99999   0.14311   0.50000
  0.43389   0.14379   0.50000
  0.85620   0.56611   0.50000
  0.99999   0.28282   0.50000
  0.54343   0.08686   0.50000
  0.85688   0.85688   0.50000
  0.85620   0.29009   0.50000
  0.43388   0.29009   0.50000
  0.71717   0.71717   0.50000
  0.08686   0.54343   0.50000
  0.85688   0.99999   0.50000
  0.29009   0.43389   0.50000
  0.29009   0.85620   0.50000
  0.71717   0.99999   0.50000
  0.45656   0.54343   0.50000
  0.00000   0.85688   0.50000
  0.56610   0.85620   0.50000
  0.14379   0.43388   0.50000
  0.00000   0.71717   0.50000
  0.45656   0.91313   0.50000
  0.14311   0.14311   0.50000
  0.14379   0.70990   0.50000
  0.56611   0.70990   0.50000
  0.28282   0.28282   0.50000
```

New structure 2:

```
    1.00000
      7.20544     -4.16006      0.00000
      0.00000      8.32013     -0.00000
     -0.00000      0.00000     11.18677
    C
```

22
Direct

0.98533	0.67638	0.50000
0.15791	0.48882	0.50000
0.66541	0.16713	0.50000
0.15791	0.66909	0.50000
0.69105	0.67639	0.50000
0.90028	0.09971	0.50000
0.83286	0.33457	0.50000
0.32361	0.30895	0.50000
0.51117	0.66909	0.50000
0.83286	0.49828	0.50000
0.33090	0.48882	0.50000
0.32360	0.01466	0.50000
0.90028	0.80057	0.50000
0.66542	0.49828	0.50000
0.69104	0.01466	0.50000
0.33090	0.84208	0.50000
0.50171	0.33458	0.50000
0.51117	0.84208	0.50000
0.98533	0.30894	0.50000
0.19942	0.09971	0.50000
0.50171	0.16713	0.50000
0.00000	0.00000	0.50000

New structure 3.

1.00000

9.27898	-5.35722	-0.00000
-0.00000	10.71444	0.00000
-0.00000	0.00000	8.71084

C
36
Direct

0.03457	0.28160	0.50000
0.43664	0.50908	0.50000
0.70162	0.61500	0.50000
0.12244	0.98219	0.50000
0.35189	0.81150	0.50000
0.59356	0.77037	0.50000
0.71839	0.75297	0.50000
0.49091	0.92756	0.50000
0.38499	0.08662	0.50000
0.01780	0.14024	0.50000
0.18849	0.54038	0.50000
0.22962	0.82318	0.50000
0.24702	0.96542	0.50000
0.07243	0.56335	0.50000
0.91337	0.29837	0.50000

0.85975 0.87755 0.50000
0.45961 0.64810 0.50000
0.17681 0.40643 0.50000
0.96542 0.71839 0.50000
0.56335 0.49091 0.50000
0.29837 0.38499 0.50000
0.87755 0.01780 0.50000
0.64810 0.18849 0.50000
0.40643 0.22962 0.50000
0.28160 0.24702 0.50000
0.50908 0.07243 0.50000
0.61500 0.91337 0.50000
0.98219 0.85975 0.50000
0.81150 0.45961 0.50000
0.77037 0.17681 0.50000
0.75297 0.03457 0.50000
0.92756 0.43664 0.50000
0.08662 0.70162 0.50000
0.14024 0.12244 0.50000
0.54038 0.35189 0.50000
0.82318 0.59356 0.50000

New structure 4.

```
   1.00000
     9.76437   -5.63746    0.00000
    -0.00000   11.27493   -0.00000
     0.00000    0.00000   10.58493
   C
   36
Direct
  0.12656  0.74488  0.50000
  0.92387  0.52608  0.50000
  0.25511  0.38167  0.50000
  0.47391  0.39779  0.50000
  0.61832  0.87343  0.50000
  0.60220  0.07612  0.50000
  0.87343  0.25511  0.50000
  0.07612  0.47391  0.50000
  0.74488  0.61832  0.50000
  0.52608  0.60220  0.50000
  0.38167  0.12656  0.50000
  0.39779  0.92387  0.50000
  0.25511  0.87343  0.50000
  0.47391  0.07612  0.50000
  0.61832  0.74488  0.50000
  0.60220  0.52608  0.50000
  0.12656  0.38167  0.50000
  0.92387  0.39779  0.50000
```

0.74488 0.12656 0.50000
0.52608 0.92387 0.50000
0.38167 0.25511 0.50000
0.39779 0.47391 0.50000
0.87343 0.61832 0.50000
0.07612 0.60220 0.50000
0.87418 0.87418 0.50000
0.75110 0.75110 0.50000
0.12581 0.00000 0.50000
0.24889 -0.00000 0.50000
0.00000 0.12581 0.50000
0.00000 0.24889 0.50000
0.12581 0.12581 0.50000
0.24889 0.24889 0.50000
0.87418 -0.00000 0.50000
0.75110 0.00000 0.50000
0.00000 0.87418 0.50000
0.00000 0.75110 0.50000

New structure 5.

```
 1.00000
   8.53061   -4.92515    0.00000
   0.00000    9.85030   -0.00000
   0.00000    0.00000    9.89811
 C
 30
Direct
 0.08629 0.66606 0.50000
 0.66799 0.83406 0.50000
 0.42022 0.33381 0.50000
 0.08396 0.91592 0.50000
 0.58150 0.16300 0.50000
 0.33393 0.42023 0.50000
 0.16593 0.83393 0.50000
 0.66618 0.08640 0.50000
 0.08407 0.16803 0.50000
 0.83699 0.41850 0.50000
 0.57976 0.91370 0.50000
 0.16606 0.33200 0.50000
 0.91359 0.57977 0.50000
 0.83196 0.91603 0.50000
 0.58149 0.41849 0.50000
 0.91370 0.33393 0.50000
 0.33200 0.16593 0.50000
 0.57977 0.66618 0.50000
 0.91603 0.08407 0.50000
 0.41849 0.83699 0.50000
 0.66606 0.57976 0.50000
```

0.83406	0.16606	0.50000
0.33381	0.91359	0.50000
0.91592	0.83196	0.50000
0.16300	0.58149	0.50000
0.42023	0.08629	0.50000
0.83393	0.66799	0.50000
0.08640	0.42022	0.50000
0.16803	0.08396	0.50000
0.41850	0.58150	0.50000

New structure 6.

1.00000

9.48756	-5.47765	0.00000
-0.00000	10.95530	-0.00000
0.00000	-0.00000	8.33203

C

36

Direct

0.38230	0.12259	0.50000
0.62750	0.16243	0.50000
0.08233	0.48879	0.50000
0.60404	0.86173	0.50000
0.87740	0.25970	0.50000
0.83756	0.46506	0.50000
0.51120	0.59354	0.50000
0.13826	0.74231	0.50000
0.74029	0.61769	0.50000
0.53493	0.37249	0.50000
0.40645	0.91766	0.50000
0.25768	0.39595	0.50000
0.12259	0.38230	0.50000
0.16243	0.62750	0.50000
0.48879	0.08233	0.50000
0.86173	0.60404	0.50000
0.25970	0.87740	0.50000
0.46506	0.83756	0.50000
0.59354	0.51120	0.50000
0.74231	0.13826	0.50000
0.61769	0.74029	0.50000
0.37249	0.53493	0.50000
0.91766	0.40645	0.50000
0.39595	0.25768	0.50000
-0.00000	0.86762	0.50000
0.13237	0.13237	0.50000
0.86762	0.00000	0.50000
0.12595	0.00000	0.50000
-0.00000	0.12595	0.50000
0.87404	0.87404	0.50000

0.26602 0.26602 0.50000
0.74842 0.74842 0.50000
0.73397 0.00000 0.50000
0.25157 0.00000 0.50000
-0.00000 0.73397 0.50000
-0.00000 0.25157 0.50000

New structure 7.

1.00000
6.27183 -3.62104 0.00000
-0.00000 7.24209 -0.00000
0.00000 -0.00000 9.88409
C
16
Direct
0.72927 0.45855 0.50000
0.44287 0.55712 0.50000
0.26529 0.93812 0.50000
0.06187 0.73469 0.50000
0.32081 0.16040 0.50000
0.54144 0.27071 0.50000
0.44287 0.88575 0.50000
0.06187 0.32717 0.50000
0.26530 0.32717 0.50000
0.83959 0.16040 0.50000
0.72928 0.27072 0.50000
0.11424 0.55712 0.50000
0.67282 0.73470 0.50000
0.67282 0.93812 0.50000
0.83959 0.67918 0.50000
0.33333 0.66666 0.50000

New structure 8.

1.00000
9.33416 0.00000 0.00000
-0.00000 9.33416 0.00000
0.00000 0.00000 8.23617
C
32
Direct
0.21473 0.71697 0.50000
0.02104 0.19917 0.50000
0.97931 0.64631 0.50000
0.84518 0.87219 0.50000
0.44144 0.85631 0.50000
0.87775 0.40764 0.50000
0.44341 0.59360 0.50000

0.27966	0.36670	0.50000
0.78526	0.28302	0.50000
0.97895	0.80082	0.50000
0.02068	0.35368	0.50000
0.15481	0.12780	0.50000
0.55855	0.14368	0.50000
0.12224	0.59235	0.50000
0.55658	0.40639	0.50000
0.72033	0.63329	0.50000
0.28302	0.21473	0.50000
0.80082	0.02104	0.50000
0.35368	0.97931	0.50000
0.12780	0.84518	0.50000
0.14368	0.44144	0.50000
0.59235	0.87775	0.50000
0.40639	0.44341	0.50000
0.63329	0.27966	0.50000
0.71697	0.78526	0.50000
0.19917	0.97895	0.50000
0.64631	0.02068	0.50000
0.87219	0.15481	0.50000
0.85631	0.55855	0.50000
0.40764	0.12224	0.50000
0.59360	0.55658	0.50000
0.36670	0.72033	0.50000

New structure 9.

1.00000

9.93068	0.00000	0.00000
-0.00000	9.93068	0.00000
-0.00000	0.00000	7.97982

C

36

Direct

0.32537	0.06881	0.50000
0.32701	0.21417	0.50000
0.02779	0.43306	0.50000
0.18374	0.03144	0.50000
0.31314	0.45052	0.50000
0.10215	0.15056	0.50000
0.59126	0.66670	0.50000
0.82850	0.60422	0.50000
0.19273	0.25754	0.50000
0.67462	0.93118	0.50000
0.67298	0.78582	0.50000
0.97220	0.56693	0.50000
0.81625	0.96855	0.50000
0.68685	0.54947	0.50000

0.89784   0.84943   0.50000
0.40873   0.33329   0.50000
0.17149   0.39577   0.50000
0.80726   0.74245   0.50000
0.93118   0.32537   0.50000
0.78582   0.32701   0.50000
0.56693   0.02779   0.50000
0.96855   0.18374   0.50000
0.54947   0.31314   0.50000
0.84943   0.10215   0.50000
0.33329   0.59126   0.50000
0.39577   0.82850   0.50000
0.74245   0.19273   0.50000
0.06881   0.67462   0.50000
0.21417   0.67298   0.50000
0.43306   0.97220   0.50000
0.03144   0.81625   0.50000
0.45052   0.68685   0.50000
0.15056   0.89784   0.50000
0.66670   0.40873   0.50000
0.60422   0.17149   0.50000
0.25754   0.80726   0.50000

New structure 10.

```
   1.00000
     8.77212       0.00000       0.00000
    -0.00000       8.77212       0.00000
     0.00000       0.00000       8.46549
   C
   28
Direct
  0.29083    0.41223    0.50000
  0.86112    0.34550    0.50000
  0.70917    0.41223    0.50000
  0.34550    0.86112    0.50000
  0.91656    0.19258    0.50000
  0.19258    0.91656    0.50000
  0.57445    0.00000    0.50000
  0.70916    0.58776    0.50000
  0.13887    0.65449    0.50000
  0.29082    0.58776    0.50000
  0.65449    0.13887    0.50000
  0.08343    0.80741    0.50000
  0.80741    0.08343    0.50000
  0.42554 -0.00000    0.50000
  0.58776    0.29083    0.50000
  0.65449    0.86112    0.50000
  0.58776    0.70917    0.50000
```

0.13887	0.34550	0.50000
0.80741	0.91656	0.50000
0.08343	0.19258	0.50000
-0.00000	0.57445	0.50000
0.41223	0.70916	0.50000
0.34550	0.13887	0.50000
0.41223	0.29082	0.50000
0.86112	0.65449	0.50000
0.19258	0.08343	0.50000
0.91656	0.80741	0.50000
0.00000	0.42554	0.50000

New structure 11.

1.00000

10.03986	-0.00000	0.00000
0.00000	10.03986	-0.00000
0.00000	0.00000	7.80720

C

36

Direct

0.34854	0.96229	0.50000
0.39867	0.09126	0.50000
0.12586	0.46039	0.50000
0.19394	0.96450	0.50000
0.34948	0.53908	0.50000
0.68579	0.32706	0.50000
0.15887	0.11127	0.50000
0.73910	0.56302	0.50000
0.28869	0.18090	0.50000
0.65145	0.03770	0.50000
0.60132	0.90873	0.50000
0.87413	0.53960	0.50000
0.80605	0.03549	0.50000
0.65051	0.46091	0.50000
0.31420	0.67293	0.50000
0.84112	0.88872	0.50000
0.26089	0.43697	0.50000
0.71130	0.81909	0.50000
0.03770	0.34854	0.50000
0.90873	0.39867	0.50000
0.53960	0.12586	0.50000
0.03549	0.19394	0.50000
0.46091	0.34948	0.50000
0.67293	0.68579	0.50000
0.88872	0.15887	0.50000
0.43697	0.73910	0.50000
0.81909	0.28869	0.50000
0.96229	0.65145	0.50000

0.09126	0.60132	0.50000
0.46039	0.87413	0.50000
0.96450	0.80605	0.50000
0.53908	0.65051	0.50000
0.32706	0.31420	0.50000
0.11127	0.84112	0.50000
0.56302	0.26089	0.50000
0.18090	0.71130	0.50000

New structure 12.

1.00000

9.55880	0.00000	-0.00000
-0.00000	9.55880	0.00000
0.00000	-0.00000	10.66403

C

29

Direct

0.42436	0.24161	0.50000
0.18031	0.29011	0.50000
0.07222	0.81634	0.50000
0.57563	0.75838	0.50000
0.81968	0.70988	0.50000
0.92777	0.18365	0.50000
0.75838	0.42436	0.50000
0.70988	0.18031	0.50000
0.18365	0.07222	0.50000
0.24161	0.57563	0.50000
0.29011	0.81968	0.50000
0.81634	0.92777	0.50000
0.57563	0.24161	0.50000
0.81968	0.29011	0.50000
0.92777	0.81634	0.50000
0.42436	0.75838	0.50000
0.18031	0.70988	0.50000
0.07222	0.18365	0.50000
0.24161	0.42436	0.50000
0.29011	0.18031	0.50000
0.81634	0.07222	0.50000
0.75838	0.57563	0.50000
0.70988	0.81968	0.50000
0.18365	0.92777	0.50000
0.61293	0.61293	0.50000
0.38706	0.38706	0.50000
0.38706	0.61293	0.50000
0.61293	0.38706	0.50000
0.50000	0.50000	0.50000

New structure 13.

1.00000

12.00868	-0.00000	-0.00000
-0.00000	12.00868	-0.00000
0.00000	0.00000	7.03331

C

32

Direct

0.34171	0.14686	0.50000
0.14365	0.22875	0.50000
0.05676	0.85660	0.50000
0.55498	0.15023	0.50000
0.65828	0.85313	0.50000
0.85634	0.77124	0.50000
0.94323	0.14339	0.50000
0.44501	0.84976	0.50000
0.85313	0.34171	0.50000
0.77124	0.14365	0.50000
0.14339	0.05676	0.50000
0.84976	0.55498	0.50000
0.14686	0.65828	0.50000
0.22875	0.85634	0.50000
0.85660	0.94323	0.50000
0.15023	0.44501	0.50000
0.65828	0.14686	0.50000
0.85634	0.22875	0.50000
0.94323	0.85660	0.50000
0.44501	0.15023	0.50000
0.34171	0.85313	0.50000
0.14365	0.77124	0.50000
0.05676	0.14339	0.50000
0.55498	0.84976	0.50000
0.14686	0.34171	0.50000
0.22875	0.14365	0.50000
0.85660	0.05676	0.50000
0.15023	0.55498	0.50000
0.85313	0.65828	0.50000
0.77124	0.85634	0.50000
0.14339	0.94323	0.50000
0.84976	0.44501	0.50000

New structure 14.

1.00000

8.03891	0.00000	-0.00000
-0.00000	8.03891	-0.00000
0.00000	-0.00000	8.63714

C

21

Direct
0.50000 0.28223 0.50000
0.50000 0.71776 0.50000
0.71776 0.50000 0.50000
0.28223 0.50000 0.50000
0.34151 0.78269 0.50000
0.08500 0.78382 0.50000
0.65848 0.21730 0.50000
0.91499 0.21617 0.50000
0.21730 0.34151 0.50000
0.21617 0.08500 0.50000
0.78269 0.65848 0.50000
0.78382 0.91499 0.50000
0.65848 0.78269 0.50000
0.91499 0.78382 0.50000
0.34151 0.21730 0.50000
0.08500 0.21617 0.50000
0.78269 0.34151 0.50000
0.78382 0.08500 0.50000
0.21730 0.65848 0.50000
0.21617 0.91499 0.50000
0.50000 0.50000 0.50000

New structure 15.

1.00000
9.73535 -0.00000 -0.00000
0.00000 9.73535 0.00000
0.00000 0.00000 8.68187
C
33
Direct
0.57759 0.37296 0.50000
0.84733 0.81368 0.50000
0.16143 0.57457 0.50000
0.06418 0.46477 0.50000
0.31328 0.94699 0.50000
0.45084 0.29703 0.50000
0.89479 0.28983 0.50000
0.06345 0.08009 0.50000
0.42240 0.62703 0.50000
0.15266 0.18631 0.50000
0.83856 0.42542 0.50000
0.93581 0.53522 0.50000
0.68671 0.05300 0.50000
0.54915 0.70296 0.50000
0.10520 0.71016 0.50000
0.93654 0.91990 0.50000
0.62703 0.57759 0.50000

0.18631	0.84733	0.50000
0.42542	0.16143	0.50000
0.53522	0.06418	0.50000
0.05300	0.31328	0.50000
0.70296	0.45084	0.50000
0.71016	0.89479	0.50000
0.91990	0.06345	0.50000
0.37296	0.42240	0.50000
0.81368	0.15266	0.50000
0.57457	0.83856	0.50000
0.46477	0.93581	0.50000
0.94699	0.68671	0.50000
0.29703	0.54915	0.50000
0.28983	0.10520	0.50000
0.08009	0.93654	0.50000
0.50000	0.50000	0.50000

New structure 16.

1.00000

10.25296	0.00000	0.00000
0.00000	10.25296	-0.00000
0.00000	0.00000	7.82740

C

36

Direct

0.76370	0.35256	0.50000
0.56397	0.34142	0.50000
0.11600	0.41867	0.50000
0.23629	0.64743	0.50000
0.43602	0.65857	0.50000
0.88399	0.58132	0.50000
0.64743	0.76370	0.50000
0.65857	0.56397	0.50000
0.58132	0.11600	0.50000
0.35256	0.23629	0.50000
0.34142	0.43602	0.50000
0.41867	0.88399	0.50000
0.23629	0.35256	0.50000
0.43602	0.34142	0.50000
0.88399	0.41867	0.50000
0.76370	0.64743	0.50000
0.56397	0.65857	0.50000
0.11600	0.58132	0.50000
0.35256	0.76370	0.50000
0.34142	0.56397	0.50000
0.41867	0.11600	0.50000
0.64743	0.23629	0.50000
0.65857	0.43602	0.50000

0.58132	0.88399	0.50000
-0.00000	0.65517	0.50000
-0.00000	0.34482	0.50000
0.34482	-0.00000	0.50000
0.65517	-0.00000	0.50000
0.80754	-0.00000	0.50000
0.19245	-0.00000	0.50000
-0.00000	0.80754	0.50000
-0.00000	0.19245	0.50000
0.88841	0.88841	0.50000
0.11158	0.11158	0.50000
0.11158	0.88841	0.50000
0.88841	0.11158	0.50000
